# Inferring the sequence of brain volume changes in progressive supranuclear palsy using MRI

**DOI:** 10.1093/braincomms/fcac113

**Published:** 2022-05-12

**Authors:** Nicolai Franzmeier, Günter U. Höglinger

**Affiliations:** 1 Institute for Stroke and Dementia Research, Klinikum der Universität München, Ludwig-Maximilians-Universität LMU, Munich, Germany; 2 German Center for Neurodegenerative Diseases (DZNE), Munich, Germany; 3 Department of Neurology, Hannover Medical School, Hannover, Germany

## Abstract

This scientific commentary refers to ‘A data-driven model of brain volume changes in progressive supranuclear palsy’ by Scotton *et al*. (https://doi.org/10.1093/braincomms/fcac098)

**This scientific commentary refers to ‘A data-driven model of brain volume changes in progressive supranuclear palsy’ by Scotton *et al*. (https://doi.org/10.1093/braincomms/fcac098)**

Progressive supranuclear palsy (PSP) is a neurodegenerative disease defined by the aggregation and spread of tau protein isoforms with four microtubule-binding repeat domains (4R-tau) in neurons, astrocytes and oligodendrocytes of the central nervous system.^1^ Most frequently, PSP presents clinically with a combination of supranuclear gaze palsy and postural instability, commonly referred to as Richardson’s syndrome (PSP-RS). However, PSP pathology can also manifest in a broad spectrum of variant clinical phenotypes (vPSP). Both PSP-RS and vPSP phenotypes take a chronic progressive and ultimately fatal disease course, leading to death after a mean of 6–7 years. Clinical progression appears to be determined by the progressive spreading of 4R-tau pathology within the brain. To better understand the spreading pattern of 4R-tau pathology, we have recently analysed the brains of *N* = 81 PSP-RS patients and proposed a neuropathological staging system with six sequential stages of PSP-RS, starting in the pallido-nigro-luysian system and spreading rostrally via striatum and amygdala to the cerebral cortex (frontal > temporo-parietal > occipital), and caudally to the medulla oblongata, pons and cerebellum.^2^

Given that PSP is a progressive neurodegenerative disease, there is a great need for an objective tracking system of disease progression in living patients for multiple reasons, including staging the clinical and pathophysiological disease status, predicting future disease trajectories, and particularly for quantifying the progression of pathological brain changes in the context of therapeutic trials. To this end, past studies have mainly employed structural MRI-based volumetry of specific cerebral regions that show early atrophy in PSP patients (e.g. midbrain).^3,4^

In this issue of Brain Communications, Scotton *et al*.^5^ undertook a more holistic approach to understand PSP progression beyond single regions that are particularly vulnerable to PSP pathology. Specifically, they used structural MRI to estimate the sequence in which brain atrophy progresses in PSP patients using a probabilistic event-based modelling (EBM) approach. In a large sample of cross-sectional structural MRI data from PSP-RS patients (*N* = 356) and healthy controls (*N* = 289), they quantified the volumes of 19 cortical and subcortical regions of interest which were selected based on our previously established neuropathological staging system for 4R-tau deposition in PSP.^2^ Using a fully data-driven mathematical approach, they have established a ranked sequence of atrophy progression across these brain regions, thereby inferring 19 sequential disease stages. Using longitudinal MRI data from a subset of PSP-RS patients (*N* = 275), they were able to demonstrate that almost all patients progressed within the 12-months follow-up period within this MRI-based staging system. Importantly, they report a linear correlation between the patients’ MRI-inferred disease stage and clinical scores on the PSP rating scale, i.e. a generally accepted clinical measure of PSP disease severity. Thereby, the authors conclude that their atrophy-based MRI staging system may aid in stratifying patients on entry into clinical trials, to improve cohort homogeneity, to track disease progression in the context of therapeutic trials and potentially to increase the power to detect a treatment effect.

The work of Scotton *et al*.^5^ presents a major advance for clinical PSP research, since it opens the possibility to track and stage disease progression by objective MRI-based measures in living patients. The sheer number and quality of the included MRI data provide solid grounds as basis for reliable conclusions, yet, the current work also poses further questions for future research.

First, the sequence of events in our pathologically defined 4R-tau staging system^2^ and the MRI staging system proposed by Scotton *et al*.^5^ in this issue of Brain Communications follow similar general patterns, but show particular distinctions though. For example, the medulla oblongata is the first of 19 stages on MRI, but second of six stages in pathology; globus pallidus is the eighth of 19 stages on MRI, but the first of six stages in pathology. Thus, the tauopathy findings appear not to translate directly into atrophy. The differential affection of neurons, astrocytes and oligodendrogytes^2^ might contribute to this discrepancy, as well as atrophy in brain areas connected to sites of tau deposits due to indirect effects.

Secondly, it remains to be shown by future studies, whether the power to detect change with time or within an intervention study is superior when using the EBM model^5^ or a classical volumetric change analysis.^3^

Thirdly, it will be very interesting to study the evolution of atrophy in the EBM model in vPSP patients. Our neuropathological data suggest that tau pathology rather uniformly emerges first in neurons in the pallido-nigro-luysian system in PSP patients, with clinical subtypes being distinguished by distinct downstream tau spreading patterns along different circuits.^2^ This concept is supported by our most recent observation that tau pathology in PSP appears indeed to spread along neuronal connectivity pathways.^6^ It remains to be elucidated prospectively which factors predispose for the spreading routes occurring in individual patients, thereby leading to different clinical PSP variants.

Finally, it will be highly interesting to study if second-generation tau-PET studies that allow *in vivo* assessment of 4R-tau levels in PSP patients^6,7^ will demonstrate identical or differential pattern of subsequent involvement of brain structures in PSP. Communalities and distinctions between these imaging modalities will be highly informative about how the progressive spreading of tau pathology and volume loss as assessed on structural MRI relate to one another.

Taken together, the authors are to be applauded for advancing the field by joining high-quality data from prior studies of multiple sources to be subjected to high-end analytical methods. The novel model appears to present a relevant tool to track disease progression in PSP with relevance for future mechanistic and interventional studies.

## Data Availability

Data sharing is not applicable to this article as no new data were created or analysed.
